# High Temporal Variability in Late Blight Pathogen Diversity, Virulence, and Fungicide Resistance in Potato Breeding Fields: Results from a Long-Term Monitoring Study

**DOI:** 10.3390/plants11182426

**Published:** 2022-09-16

**Authors:** Eve Runno-Paurson, Helina Nassar, Terje Tähtjärv, Viacheslav Eremeev, Merili Hansen, Ülo Niinemets

**Affiliations:** 1 Institute of Agricultural and Environmental Sciences, Estonian University of Life Sciences, Kreutzwaldi 1, 51006 Tartu, Estonia; 2 Estonian Crop Research Institute, J. Aamisepa 1, 48309 Jõgeva, Estonia

**Keywords:** mating type, metalaxyl, virulence, population changes, *Phytophthora infestans*, potato cultivars

## Abstract

Long-term site-specific studies describing changes in the phenotypic variability of *Phytophthora infestans* populations allow quantitative predictions of pathogen spread and possible outbreaks of epidemics, and provide key input for regional resistance breeding programs. Late blight samples were collected from potato (*Solanum tuberosum*) breeding fields in Estonia during a twelve-year study period between 2001 and 2014. In total, 207 isolates were assessed for mating type and 235 isolates for metalaxyl resistance and 251 isolates for virulence factors. The frequency of mating types strongly fluctuated across the years, whereas the later period of 2010–2014 was dominated by the A2 mating. Despite fluctuations, both mating types were recorded in the same fields in most years, indicating sustained sexual reproduction of *P. infestans* with oospore production. Metalaxyl-resistant and intermediately resistant strains dominated in the first years of study, but with the progression of the study, metalaxyl-sensitive isolates became dominant, reaching up to 88%. Racial diversity, characterized by normalized Shannon diversity index decreased in time, varying from 1.00 in 2003 to 0.43 in 2013. The frequency of several virulence factors changed in a time-dependent manner, with R2 increasing and R6, R8, and R9 decreasing in time. Potato cultivar resistance background did not influence the frequency of *P. infestans* mating type, response to metalaxyl, and racial diversity. However, the diversity index decreased in time among isolates collected from resistant and susceptible cultivars, and remained at a high level in moderately resistant cultivars. These data demonstrate major time-dependent changes in racial diversity, fungicide resistance, and virulence factors in *P. infestans*, consistent with alterations in the control strategies and popularity of potato cultivars with different resistance.

## 1. Introduction

Potato (*Solanum tuberosum*) late blight caused by the oomycete pathogen *Phytophthora infestans* is a serious problem for potato growers worldwide, and its first emergence in European potato fields was in the 1840s. Late blight is a re-emerging and persistent disease [1,2], causing substantial economic losses [3]. It is extraordinarily virulent and adaptable, reflecting continuous evolution of new lines [4,5,6,7]. In the last two decades, late blight pathogen populations underwent fast and sudden genetic changes due to within-population changes in genotype frequencies as well as crossings among populations, causing frequent genotype shifts throughout potato cultivation areas in Europe, North America, Asia, and Africa [4,8,9,10,11,12,13,14,15,16,17,18].

Due to increased adaptability, late blight is able to destroy potato foliage even under unfavorable conditions for the pathogen before the end of the growing season [19,20]. Hence the use of fungicides plays a key role in late blight integrated control strategies. In Europe, an average of 7–15 fungicide treatments per growing season are applied, depending on weather conditions, disease pressure, and cultivar [21,22,23,24]. In several European countries, intensive late blight control management with a weekly schedule of fungicide spraying are implemented [25]. Late blight infections start nowadays earlier in the growing season [6], and under favorable conditions for the pathogen, farmers are forced to spray potato foliage with fungicides even every three to five days to control the disease [19,24]. Thus, in very severe blight years, fungicides are applied up to 20–25 times during the growing season [21,22,26].

A major concern is the increasing fungicide resistance of late blight [24], and there is a need to use several active ingredients due to the insensitivity of novel strains to common fungicides [23]. Metalaxyl-resistant isolates of *P. infestans* were reported already in the early 1980s in Europe [27,28], but the loss of efficacy of metalaxyl under practical field conditions was not always scientifically proven, especially in Northern Europe, e.g., in Finland [29]. Similarly, the broad-spectrum fungicide product Ridomil Gold MZ 68 WG (Syngenta; metalaxyl and mancozeb as active ingredients) is widely used as an effective fungicide by potato growers in Estonia [30]; however, the approval for its EU use expired in January 2022. Some tolerance of *P. infestans* to another common fungicide propamocarb HCl was found, but no signs of resistance in *P. infestans* or failures in late blight control were reported [21,29,31,32]. New genotypes of *P. infestans* caused reduced efficacy of fluazinam (product name Shirlan 500 SC), which was a widely used tool for tuber blight control [23]. In particular, fluazinam is not effective for the EU_37_A2 strain, a new widespread *P. infestans* genotype in European potato fields [33].

Use of cultivar resistance in potato late blight control became more important due to the increased severity of late blight infections, increased pathogen diversity, and resultant adaptability and fungicide resistance development. In light of the European Green Deal, it is further necessary to use more sustainable control practices and reduce the agrochemical input, and to overall redesign the production systems [34]. However, this does not reflect the real situation in potato fields, as a large portion of cultivars grown in Europe do not comprise any resistance genes and are susceptible or even very susceptible to late blight [19,21,35]. Thus, it is highly challenging to find late blight-resistant cultivars with persistent field resistance, adapted to local field conditions, and with the traits that correspond to producer’s demands [21]. Traditional breeding methods, such as parental and progeny selection, are relatively slow, and relying only on these methods would imply that late blight resistance would inevitably lag behind the spread of new late blight lineages. Application of new methods can improve the speed and efficiency of future breeding efforts that aim to simultaneously increase crop disease resistance and yield and improve taste; these new approaches include effective combinations of resistance genes [36], use of diagnostic DNA markers for yield optimization [37], and improvement of taste to increase potato consumption [38].

Until the early 1970s, in Estonia, the main yield-limiting factor for potato growth was drought in some exceptionally dry summers. Appearance of new more virulent late blight strains was the game changer, and the first early potato late blight outbreaks were observed in Estonia at Jõgeva potato breeding fields in 1972 [39]. Thus, since the early 1970s, the new target in potato breeding programs was to improve gene bank with resistant breeding material and intensive breeding for the diversification of late blight-resistant cultivars. Nowadays, improved phytopathogenic profile (resistance for several pathogens, including *P. infestans*) and early tuber maturity are the main targets in Estonian potato breeding programs. However, a special late blight resistance breeding program ended in 2012 [40]. Local breeding has major regional relevance, especially for Nordic countries, because no major potato breeding companies from Western or Central Europe develop cultivars bred for cultivation in northern latitudes [41]. Although the Western and Central European cultivars might have desirable traits, they may not cope well with local conditions outside their breeding area. For example, the Dutch cultivar Toluca has early medium maturation and high late blight resistance in the Netherlands, but it did not thrive well under drought conditions in the Northern Baltics and could not exhibit late blight resistance under these conditions [19,35].

Late blight is constituting a direct threat to potato production worldwide, and due to the sexual reproduction of the pathogen, there is ongoing rapid change in genotypes and diversification within its populations. Thus, continuous monitoring of *P. infestans* populations at global, regional, and local levels is needed for developing integrated plant protection and breeding strategies to combat the infections. The present study characterized the mating type, response to metalaxyl and virulence of *P. infestans* isolates in the Estonian potato breeding fields in Jõgeva during 12 years of study. The main aim of this long-term study was to monitor *P. infestans* populations in a location with a high variation in a host-resistance background to gain an insight into changes in *P. infestans* populations, as driven by the inflow of new lineages and host resistance. *Phytophthora infestans* isolates were collected from potato cultivars with a wide range of host resistance to potato late blight, allowing for the assessment of the impact of cultivar resistance level (resistant, moderately resistant, and susceptible) on phenotypic variation in the *P. infestans* isolates studied.

## 2. Results

The main aim for this long-term study was to detect possible temporal changes in *P. infestans* sub-populations collected from potato breeding fields during a 12-year study period (2001–2007, 2010–2014). The impact of the cultivar resistance level on the phenotypic variation in *P. infestans* isolates was also studied. For that purpose, *P. infestans* isolates were characterized for mating type (207 isolates), virulence (251 isolates), and metalaxyl response (235 isolates).

### 2.1. Mating Type

**Figure 1:**
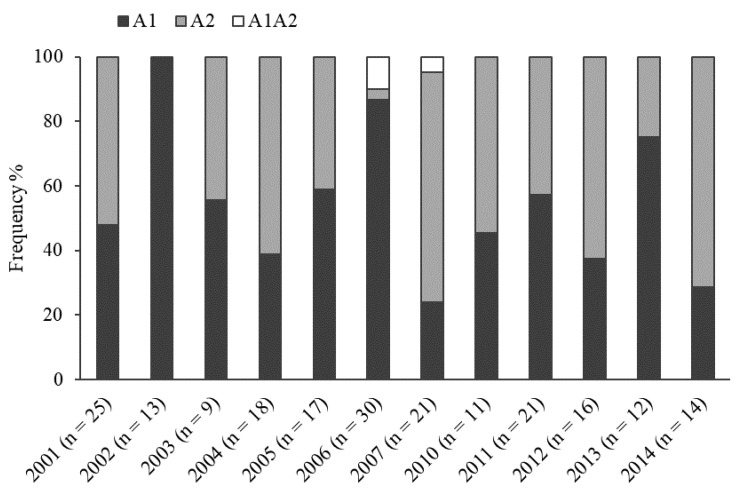
Frequency of mating types among isolates of *Phytophthora infestans* from potato (*Solanum tuberosum*) breeding fields in Estonia during 2001–2007 and 2010–2014. n is the total number of isolates assessed for mating type in each year.

Of the 207 isolates tested, 114 belonged to A1 mating type (55.1%) and 89 to A2 mating type (43.0%). Both A1 and A2 mating types were found in eleven out of twelve study years. Self-fertile isolates were observed only in 2006 and 2007, in total 1.9% of the whole population. A2 mating type was found in all years except in 2002 (Figure 1). The frequency of the A2 mating type varied between 41.2 and 71.4%, and dominated (>50%) in 2001, 2003–2005, 2007, 2010–2012, and 2014 (χ^2^ = 60.5, df = 12, *p* < 0.001 for the year effect). Considerable fluctuations in the frequency of A2 mating type were observed during 2001–2007 (Figure 1). In 2010–2012 and 2014, the frequency of the A2 mating type stayed high and stable. There were no significant differences in the frequency of A1 and A2 mating types between isolates collected from cultivars with different late blight resistance levels (χ^2^ = 1.42, df = 4, *p* = 0.84).

### 2.2. Metalaxyl Resistance

**Figure 2:**
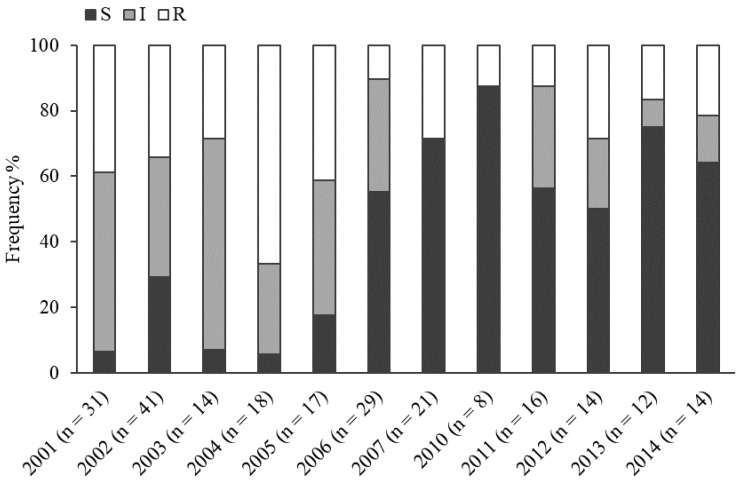
Variation in the percentage of *P. infestans* isolates with different metalaxyl-sensitivity from potato breeding fields in Estonia during 2001–2007 and 2010–2014. Metalaxyl-sensitivity as: S—sensitive; I—intermediate; and R—resistant. n is the total number of isolates assessed for metalaxyl-sensitivity in each year.

**Figure 3:**
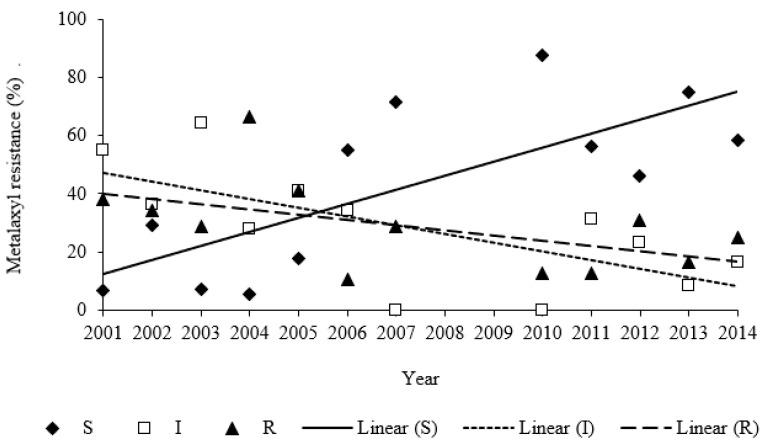
Correlations between the percentage of *P. infestans* isolates with different metalaxyl resistance and year of study in potato breeding fields in Estonia for 2001 to 2014. Metalaxyl-sensitivity as: S—sensitive; I—intermediate; and R—resistant. Data were fitted by linear regressions. S: *y* = 4.82 *x* − 9620, *r*^2^ = 0.57, *p* = 0.005; I: *y* = −3.01 *x* + 6080.4, *r*^2^ = 0.46, *p* = 0.016; R: *y* = −1.80 *x* + 3640, *r*^2^ = 0.27, and *p* = 0.087.

Of the 235 isolates tested for their response to metalaxyl, 70 isolates (29.8%) were classified as resistant, 74 isolates (31.5%) as intermediate, and 91 isolates (38.7%) as sensitive (Figure 2). The metalaxyl resistance varied between sampling years (χ^2^ = 82.2; df = 22, *p* < 0.001; Figure 2). In the period 2001–2005, metalaxyl-resistant and intermediately resistant isolates strongly prevailed, comprising together 70.7−94.5% of tested isolates (Figure 2). Metalaxyl-sensitive isolates prevailed in the years 2006–2007 and 2010–2014 with 50.0–87.5% (Figure 2). A strong interaction between the response to metalaxyl and the year was observed, implying that the proportion of sensitive isolates increased with year of study and shifted the dominance of intermediately resistant and resistant isolates (Figure 3). A marginally significant association between cultivar resistance and response to metalaxyl was found (χ^2^ = 9.10, df = 4, *p* = 0.059), whereas the frequency (27.4%) of metalaxyl-sensitivity among isolates collected from moderately *P. infestans*-resistant cultivars tended to be lower compared to susceptible (47.3%) and resistant cultivars (42.1%).

Within the metalaxyl-resistant isolates, 48 belonged to A1 and 52% to A2 mating types and 54% of metalaxyl-sensitive isolates were A1, 42% A2 mating type, and 4% A1A2 mating type. No significant association between response to metalaxyl and mating type was found (χ^2^ = 6.74, df = 4, *p* = 0.15).

### 2.3. Pathotype

Among the 251 tested isolates, all 11 known virulence factors were found (Table 1). A significant difference in the prevalence of virulence factors (R1–R11) was observed among the study years (*F*_(10, 121)_ = 49.8, *p* < 0.001). Most isolates were virulent on differentials with genes R1, R3, R4, R7, R10, and R11. Virulence factors 5 (11.0%) and 9 (13.8%) were relatively rare (Table 1), whereas factors 6 (47.2%) and 8 (42.9%), were moderately represented (Table 1). Prevalence of virulence factors 5, 6, 8, and 9 varied between collection years (factor 5: χ^2^ = 33.9, d.f. = 11, *p* < 0.001; factor 6: χ^2^ = 27.5, d.f. = 11 *p* < 0.01; factor 8: χ^2^ = 46.6, d.f. = 11 *p* < 0.001; and factor 9: χ^2^ = 36.6, d.f. = 11, *p* < 0.001). The relatively rare virulence factor 5 was found in nine years out of 12, but its occurrence was not significantly correlated with the year of study (Table 1; *r* = −0.10; *p* = 0.103). The relatively rare virulence factor 9 was found in ten years out of 12. The virulence factor 6, although infrequent, was found in each year, and factor 8 was found in all years, except for 2012 (Table 1). The frequencies of these factors, R6, R8, and R9, decreased over time (factor 6: *r* = −0.17, *p* = 0.007; factor 8: *r* = −0.19, *p* = 0.003; and factor 9: *r* = −0.23, *p* < 0.001). The incidence of virulence factor 2 varied greatly between years (χ^2^ = 35.3, d.f. = 11, *p* < 0.001) and its frequency increased over time (Table 2; *r* = −0.23, *p* < 0.001).

Factor 9 was less frequent among strains isolated from susceptible potato cultivars (5.2%) compared to moderately resistant (24.7%) and resistant (15.5%) cultivars (χ^2^ = 8.58, d.f. = 2, *p* = 0.014). The factors 7 (90.5%; χ^2^ = 6.28, d.f. = 2, *p* = 0.043) and 11 (81.0%; χ^2^ = 7.49, d.f. = 2, *p* = 0.024) were less frequent among the isolates from resistant cultivars. However, the frequency remained high in all cultivar groups (e.g., moderately resistant—factor 7: 97.4%, factor 11: 92.2%; susceptible—factor 7: 98.3%; factor 11: 93.1%). No significant differences in the frequencies of other virulence factors were observed between potato cultivar late blight resistance groups.

There was a high level of race diversity, with 86 pathotypes found among 251 tested isolates, whereas 53 phenotypes were found only once (Table S2). The number of phenotypes found only once varied strongly during the study years (11–100% from population) (Table S2). The average ± SE number of virulence factors per isolate was high (7.3 ± 0.7), and varied strongly between years, from 5.6 to 8.9 (Table 1; *F*_(11, 12)_ = 315.2, *p* < 0.001 for the year effect). The complex races dominated in 8 years out of 12. The most complex races dominated in 2001, 2005, 2006, 2007, 2013, and 2014 (Table 1). The average number of virulence factors was high for all three *P. infestans*-resistance groups: highly resistant (7.2), moderately resistant (7.3), and susceptible cultivars (7.1) (*F*_(2,26)_ = 0.52, *p* = 0.95 for group differences). During the 12 year study period, the six most common virulence races were 1.2.3.4.6.7.10.11, 1.2.3.4.7.8.10.11, 1.2.3.4.7.10.11, 1.2.3.4.6.7.8.10.11, 1.3.4.7.10.11, and 1.2.3.4.6.7.8.9.10.11 (Table S2), representing together 49% of the characterized isolates. Race composition changes occurred in every year, but the most frequent race 1.2.3.4.7.8.10.11 prevailed in 2001, 2004, 2006, 2013, and 2014. However, it was not found in year 2007 and in 2010–2012. The race 1.2.3.4.6.7.10.11 was most frequent in 2002, 2005–2007, 2010–2012, and was also found in 2001, 2004, and 2014, but it was missing in 2003 and 2006 (Figure S1 for time-dependent changes in the prevalence of most common virulence phenotypes).

**Figure 4:**
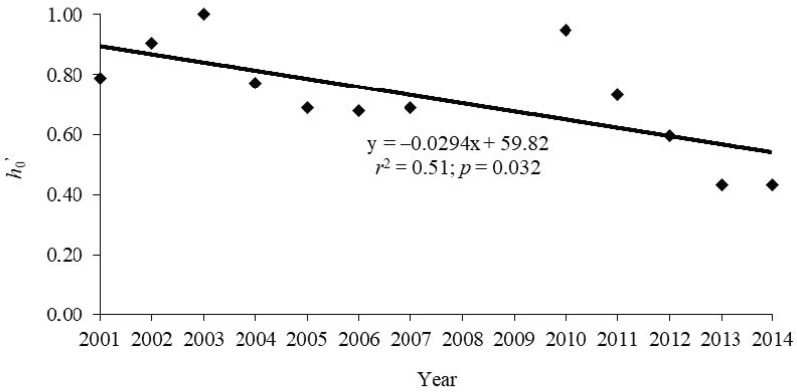
Correlation of racial diversity (normalized Shannon diversity index, *h*_0’_) of isolates of *P. infestans* with year of study in potato breeding fields in Estonia for 2001 to 2014. The data were fitted by a linear regression.

The global average normalized Shannon diversity index was 0.66. It was the highest in 2003 (1.00), 2010 (0.95), and 2002 (0.91) (Table 2; *F*_(10,11)_ = 10.7, *p* = 0.032 for the year effect). The normalized Shannon diversity index (*h*_0’_) decreased over the years (Figure 4). The average values of the Shannon diversity index for isolates collected from moderately resistant (0.89) and resistant cultivars (0.82) did not significantly differ from the average in susceptible cultivars (*h*_0’_ = 0.70; *F*_(2,26)_ = 2.11, and *p* = 0.142). The diversity index decreased in isolates collected from late blight-resistant cultivars (*r* = −0.82, *p* = 0.007) and sensitive cultivars (*r* = −0.85, *p* = 0.002) over time (Figure S2). For isolates collected from moderately resistant cultivars, the diversity index remained high through the study (*r* = 0.04, *p* = 0.92; Figure S2).

## 3. Discussion

This study examined time-dependent changes in virulence, mating type, and metalaxyl resistance of *P. infestans* isolates collected from potato breeding fields. Considerable fluctuations in the frequency of the mating types (A1 or A2) were observed over the study period. Although the mating type A2 was dominant across the whole study period, it was missing in year 2002, and its frequency was very low in 2006 (Figure 1). Nevertheless, its frequency remained steadily high for the last five years (Figure 1). Temporary fluctuations in the proportions of mating types between study years were previously observed in European populations of *P. infestans* including the Baltics [42,43,44], Finland [29,45], Poland [9,46], Czechia [47], Ireland [48], Spain [49], and the Pskov region in North-West Russia [50]. Similarly, in Moscow region of Russia, considerable fluctuations in the frequency of A1 and A2 mating types were found in a long-term *P. infestans* monitoring study, whereas the frequency of A2 mating type varied from a low to a moderate level of 3–35% in 2009–2011 and 2015–2017 to an extremely high level of 65–85% in 2012–2014 [51].

The mating type (A1 or A2) frequency depends on which genotypes at any given moment dominate the infecting *P. infestans* populations. The frequency of A2 increased in several European populations, such as the UK, where the aggressive genotype EU_13_A2 spread rapidly since its first detection in 2004 [4,12], but also in *P. infestans* populations in Central, Southern, and Western Europe [11,18,49,52,53]. The genotype EU_13_A2 was introduced later to Asia and Africa through seed potatoes imported from Europe [16,54,55]. However, in recent years, the frequency of the dominant genotype EU_13_A2 decreased among European populations of *P. infestans*, and lately (for two last seasons) was almost replaced by novel invasive lines EU_36_A2 and EU_37_A2 [53,56,57]. Strain shifts also occurred in sexually reproductive, highly diverse *P. infestans* populations in Eastern and Northern Europe; recently the invasive clonal lineage EU_41_A2 rapidly spread in Nordic areas of pathogen occurrence [53,58].

In this study, both the mating types A1 and A2 co-existed together in most of the potato fields sampled similarly to previous studies in the Baltic and Nordic countries and Eastern Europe [42,43,44,45,50,51,59,60]. All these studies highlighted the presence of genetically very diverse populations of *P. infestans* [30,46,50,61,62,63,64]. These results suggest the continuous sexual reproduction of *P. infestans* and contamination of soils with long-lived oospores, and thus indicate a direct risk of early soil infection of late blight in potato breeding fields in Estonia.

In this long-term study, we observed an increase and dominance in *P. infestans* metalaxyl-sensitive isolates among the *P. infestans* populations collected from Jõgeva breeding fields. These results concord with similar observations in several European populations of *P. infestans*, such as Baltic countries [30,42,44], the Nordic region [29,45,59], Poland [46], Belarus [65], Czechia [31], and the regions so far studied in Russia [51,65,66]. The main reason for the decrease in metalaxyl-resistant isolates is the limited use of this fungicide compared to the 1990s and 2000s [30,59,64].

In contrast, according to recent data, the proportion of metalaxyl-resistant and intermediately resistant isolates increased in Poland (2016–2020) and in Czechia (2012–2016) [60,67]. In fact, the rapidly spreading *P. infestans* genotype EU_13_A2 is metalaxyl-resistant, and the proportion of resistant isolates increased substantially since the mid-2000s in the European regions where this genotype became dominant [4,8,52,54,68,69,70]. The increase in metalaxyl resistance in EU_13_A2-dominated regions occurred despite the limited used of the fungicide (Ridomil Gold MZ 68 WG). This genotype (EU_13_A2) is not found in the Estonian and other Baltic populations of *P. infestans* [63,64].

The composition of races with a specific suite of virulence factors provides important information for site-specific potato breeding for enhanced resistance. The results of this study show that the population of *P. infestans* in Jõgeva is diverse, consists of complex races, and strongly varies among years. Considerable changes in the share of R2, R6, R8, and R9 were observed during the 12-year study (Table 1). Particularly prominent was the temporal increase in the frequency of the virulence factor R2 (Table 1). Similarly, the frequency of the virulence factor R2 was at a moderate level (32–49%) in previous population studies of *P. infestans* in Estonia [43,71], and increased in recent studies to more than 70% [30,72]. The R2 frequency is relatively high, 65–85%, in the Baltic *P. infestans* populations [30,42,44], such as in Spain (over 80%) [73] and in Russia (60–85%) [66], but at a moderate level in Czechia (61%) [47] and in Poland (40–70%) [7,9,67]. In contrast, the frequency of R2 in Finland was relatively low over the years, less than 18% on average [29]. In other Nordic countries, Denmark, Norway and Sweden, the frequency varied between 10 and 50% depending on the country [59]. *Phytophthora infestans* resistance genes R1 (*Rpi*-R1) and R2 (*Rpi*-R2) from *S. demissum* were used previously for cross-breeding in potato breeding programs in Estonia [74]. Thus, the *Rpi*-R1 gene is identified with SSR markers in most of the moderately and highly late blight-resistant cultivars ‘Ando’, ‘Mats’, ‘Olev’, ‘Piret’, ‘Reet’, ‘Tuljak’, and ‘Maret’; however, the gene *Rpi*-R2 is identified only in one breeding line ‘1182-97’ [74]. Similarly, the *Rpi*-R1 gene was present in several common susceptible and moderately resistant cultivars, such as ‘Craigs Snow White’, ‘Pentland Dell’, Picasso’, ‘Spunta’, and highly resistant cultivars ‘Cara’ and ‘Innovator’ [36], while the *Rpi*-R2 gene and *Rpi*-R2-like gene are rarer and contained in some highly late blight-resistant cultivars, such as ‘Bionica’ and ‘Innovator’ [36]. The presence of other R-genes in Estonian potato cultivars is currently unclear. Several wild *Solanum* species, such as *S. andigenum*, *S. chilense*, *S. demissum*, *S. infundibuliforme*, and *S. vernei* were used over the years in potato breeding programs, and it is likely that multiple other R-genes were introduced into the cultivated potato by crossing [19,40].

We found that pathotype shifts occurred in most monitoring years (Table S2). Nevertheless, one of the most common pathotypes, 1.2.3.4.6.7.10.11, was found in ten study years out of twelve, and the other two, 1.2.3.4.7.10.11, 1.2.3.4.7.8.10.11, prevailed in seven study years. These results are similar with other findings in Estonia for 2011–2012 [30] and for 2001–2007 [43,71]. The virulence phenotypes 1.2.3.4.6.7.10.11 and 1.2.3.4.7.10.11 also predominate in Czechian, Latvian, and Lithuanian populations [42,44,47]. The most common race in Europe, 1.3.4.7.10.11 [29,46,59,75], was found in this study only in some of the years and did not prevail during the study period. The most frequent pathotype in as long-term a study of Russian *P. infestans* populations (Leningrad region) was 1.2.3.4.5.6.7.8.10.11 [76], but this genotype was very rare in our study.

We observed a large proportion of unique pathotypes, reflecting sexual reproduction of the pathogen [30]. The average number of virulence factors (infected Black’s differentials) per isolate observed in the study area, 7.3 ± 0.7, was high. This is comparable with studies in Poland [66,77,78], Latvia, Lithuania, Russia [51,66,76], and previous works from Estonia [42,43,44], in Czechia [47]. In contrast, the number of virulence factors was lower in other populations in Estonia [30], and in Finland and in Norway [59], indicating variation in the degree of sexual reproduction in different locations. In fact, we found that race diversity characterized by the Shannon index also decreased notably over the study period. This is probably due to the fact that there were fewer isolates from *P. infestans*-resistant potato cultivars in the later years of the study; the number of resistant cultivars and breeding lines were considerably less at the end than in the beginning of the study.

The potato cultivar resistance plays an important role in the control of late blight, but does it also affect *P. infestans* populations? This aspect is less studied because the majority of potato cultivars grown in commercial fields generally does not harbor any resistance to *P. infestans* and the traditional commercial cultivars are susceptible or very susceptible to late blight [19,21]. Our study shows that cultivar resistance background did not influence the frequency of the *P. infestans* mating type, response to metalaxyl, and the race diversity. However, the diversity index decreased in time among isolates collected from resistant and susceptible cultivars, and remained at a high level in moderately resistant cultivars. Contrary to our results, Stellingwerf et al. [48] found that late blight isolates sampled from resistant potato genotypes, such as ‘Sarpo Mira’ and ‘Bionica’, were more often of the EU_13_A2 lineage than those sampled from potato cultivars with low resistance. This aggressive strain dominated European populations for many years and is a major cause of severe late blight epidemics in potato fields [24,25]. Analogously, Flier et al. [79] and Blandón-Díaz et al. [80] pointed out that *P. infestans* isolates sampled from highly resistant cultivars have more complex virulence races.

In general, breeding for highly productive, genetically homogenous, and disease-resistant cultivars of main agricultural crops does not keep pace with pathogen spread and divergence, resulting in large and devastating epidemics [81], including the emergence or re-emergence of major pathogens [82,83]. In addition, since the early 2000s, there was the pressure for commercial cultivars not only to be high-yielding, but to mature early and have more attractive tubers with smoother skin; thus, breeding for the late blight resistance was relegated to the background [40]. Targeted breeding for late blight resistance basically ended in Estonia in 2009; and since then, the breeding lines with late blight resistance are used in combination with lines with other desirable traits in breeding programs [84]. In Estonian potato breeding program, every year 60 different cross-parents are used to create 2500 combinations, and about 10% of crossings contain late blight resistance genes R1 and R2 [74]. Lack of emphasis on late blight resistance is also evident in this work; as noted above, fewer isolates were available from highly resistant cultivars at later stages of the study.

Increasing use of highly specialized fungicides is expected to lead to pathogen resistance development and subsequent loss in the efficacy of fungicides, implying a constant need for novel chemicals for plant protection [85,86]. In addition, the European Green Deal foresees a major reduction in pesticide use in European agriculture [87]. Exploiting host resistance, adaptation of landraces to new management systems, and use of decision support systems (DSS) for the selection of cultivars and application of plant protection measures are essential tools to curb fungicide use while limiting the risk to plant health and resistance development [88,89,90]. All these approaches applied together may help achieving the goals of the European Green Deal [87]. Locally bred and adapted potato cultivars are also needed to adapt to the increased frequency of drought stress episodes caused by changes in precipitation patterns and to cope with near future climate change in Northern Europe [91,92].

Given the devastating spread of *P. infestans*, finding methods to employ major resistance genes against *P. infestans* remains an important goal for potato breeding [93]. Currently, breeders isolate variants of R genes and deploy them in pyramids or stacks for obtaining durable and broad spectrum resistance against late blight in the field [94,95]. However Rakosy-Tican et al. [96] showed that the combination of somatic hybridization with the use of gene-specific markers and corresponding avirulence (*Avr*) effectors is an efficient approach for the successful introgression of late blight resistance genes into the potato gene pool. Thus, the information of the diversity of virulence phenotypes in potato breeding fields provides key input for the breeding of highly field-resistant cultivars. As *P. infestans* is now also a soil-borne pathogen, the longer crop rotations remain perhaps one of the most effective control methods for late blight in Northern European areas. However, the epidemics can end primarily by the replacement of susceptible cultivars with moderately resistant or resistant cultivars and the rapid registration of alternative fungicides [97,98,99].

## 4. Materials and Methods

### 4.1. Collection and Isolation of P. infestans Isolates

Potato leaves infected by *P. infestans* were collected from potato breeding fields at the Estonian Crop Research Institute in Jõgeva, Jõgeva County, Estonia (58°45′ N, 26°24′ E) during 12 years (2001–2007 and 2010–2014; Table 3). In total, 251 *P. infestans* isolates were analyzed. In this dataset, 180 isolates were analyzed in a previous study by Runno-Paurson et al. [100], but the host resistance aspect of potato late blight was not considered. The data were reanalyzed with new a data set of *P. infestans* from 2010 to 2014. Most isolates originated from leaves, and the tuber isolates were collected only in 2001. During the 12-year study, infected leaf samples were collected in different epidemiological phases, at the beginning of late blight infection, in mid-outbreak (1–2 weeks later) and at the end of the growing season (>3 weeks later). In the early stages of the outbreak, approximately 10–15% of the leaf area of the infected plants and less than 10% of plants were infected with late blight. In the later stages, about 20–30% of the leaf area and more than 50% of the plants were infected. Overall, the study area is characterized by high genetic diversity of the host plants, including several genotypes that have race-specific genes [101]. Samples from potato plants were collected randomly across the field. From each plant, only single-lesion leaves were taken at random, excluding any with several or no lesions. At each site, leaf samples were collected from three to twenty-two different cultivars or breeding lines with varying late blight resistance levels (Table 3 and Table S1). In total, 116 isolates were obtained from resistant cultivars and breeding lines; 81 isolates from moderately resistant cultivars and breeding lines; and 54 isolates from susceptible cultivars (Table S1). In the breeding fields, conventional agrotechnical methods were used without the application of fungicide treatment during the period 2001–2007. Due to the earlier outbreaks of late blight infestation, one-time fungicide treatments for preventive control of late blight were applied in 2010 (tattoo C; propamocarb + chlorothalonil), 2011 (Electis 75 WG; zoxamide + mancozeb) and in 2014 (Orvego; ametoctradin + dimethomorph).

Eleven to forty-one isolates were cultured in each study year (Table 1). For pure culture isolation, tubers of susceptible cultivars without known R-genes were used (Berber, Bintje). The tubers were washed and dried, sliced and flame-sterilized, and a fragment of infected leaf tissue placed between ethanol and the sterilized tuber slices. The slices were placed onto sterile Petri dishes with a moist filter paper disc on top and incubated for 6–7 days at 16 °C in a growth chamber until the mycelia grew through the slices. A small sample of mycelia from the tuber slices was transferred with a sterile needle to rye B agar. The pure cultures were preserved at 5 °C and transferred to the rye agar after every two months. All phenotypic tests were carried out in October–November of the year of isolation.

### 4.2. Phenotypic Analyses

Mating types were determined by the method described in Runno-Paurson et al. [101] for 207 isolates. The tester isolates were 90209 (A1) and 88055 (A2) as described in Hermansen et al. [75]. Isolates forming oospores on plates with the A1 mating type were registered as A2; isolates that formed oospores with the A2 mating type were registered as A1.

The resistance to metalaxyl of 171 isolates collected during 2001–2007 was tested using a modification of the floating leaflet method (leaflets in a plastic tray) [75] as described in Runno-Paurson et al. [102]. For 64 isolates collected during 2010–2014, the resistance to metalaxyl was tested with a modification of the floating leaflet method (leaf disks in Petri plates) by Runno-Paurson et al. [44].

The virulence pathotype was determined for 251 isolates with a detached leaflet using a set of Black’s differentials of potato genotypes containing resistance genes R1–R11 from *Solanum demissum* provided by the Scottish Agricultural Science Agency [102]. Laboratory procedures were as described in Runno-Paurson et al. [101]. *Phytophthora infestans* isolates from this study are preserved in pure culture at Tartu Fungal Collection (TFC) in Estonia.

### 4.3. Data Analysis

Statistical analyses were performed with SAS/STAT version 9.1 (SAS Institute Inc., Cary, NC, USA). Differences in the prevalence of the two mating types among *P. infestans* isolates between years were tested using a logistic analysis (GENMOD procedure in SAS) with a multinomial response variable (A1, A2, or both). Analogous logistic procedures were used to examine the differences in the resistance to metalaxyl (a multinomial response variable: resistant, intermediate, or sensitive) between years, and also between different mating types.

Separate logistic analyses were used to test for the differences in the prevalence of virulence against different R genes (virulent vs. non-virulent) between years, and for the dependence of mating type on race prevalence (unique vs. prevalent). Variation in virulence complexity among different years and racial diversity were analyzed with one-way ANOVA and Tukey HSD post hoc test and the differences were considered significant at *p* < 0.05.

Pathotype diversity was computed based on the Shannon (*H*_s_ = −Σ*P*_i_Ln*P*_i_) and the normalized Shannon (*h*_0’_ = −Σ*P*_i_Ln*P*_i_/lnN) indices, where *P*_i_ is the frequency of the i-th pathotype and N the sample size. *h*_0’_ gives the Shannon index as a fraction of the maximum diversity in the sample, and it ranges from 0 (single pathotype present) to 1 (each isolate in the sample has a different pathotype). This statistic provides a more representative basis for comparison when sample sizes vary [103]. Regression analyses were used to test for the time-dependent trends in normalized Shannon index values across the study years. Pearson correlation coefficients were calculated to analyze the statistical relatedness among the studied variables at significance levels *p* < 0.001, *p* < 0.01, *p* < 0.05, or *ns* (no significant).

## Figures and Tables

**Table 1:** Race frequencies among isolates, average number of virulence factors per isolate and average ± SE number of isolates in different years in *P. infestans* collected from breeding fields in Estonia during 2001–2007 and 2010–2014.

Year	Virulence to Potato Resistance Gene (%)											Number of Virulence Factors per Isolate	Number of Tested Isolates
	R1	R2	R3	R4	R5	R6	R7	R8	R9	R10	R11		
2001	91	71	83	97	14	69	100	54	46	97	100	8.2 ^c^*	35
2002	93	46	100	83	7	61	98	44	20	71	49	6.7 ^b^	41
2003	64	50	86	71	7	50	64	79	21	86	86	6.6 ^b^	14
2004	84	63	89	84	5	32	100	42	11	95	100	7.1 ^bc^	19
2005	100	94	100	100	56	67	100	56	17	100	100	8.9 ^d^	18
2006	90	60	83	93	7	53	97	47	3	83	100	7.2 ^bc^	30
2007	100	78	91	96	17	35	100	43	0	100	96	7.6 ^bc^	23
2010	64	45	91	64	9	36	82	27	9	73	64	5.6 ^a^	11
2011	81	81	81	71	10	62	76	14	10	86	81	6.6 ^b^	21
2012	87	93	67	87	0	60	100	0	13	87	100	6.9 ^b^	15
2013	100	100	100	100	0	33	100	83	0	100	100	8.2 ^c^	12
2014	100	83	100	100	0	8	100	25	17	100	100	7.3 ^bc^	12
Total	89	69	89	88	12	51	94	43	16	88	87	7.3 ± 0.7	251

* Values with different superscripts differ significantly from each other at *p* < 0.05 (one-way ANOVA followed by Tukey HSD test).

**Table 2:** Racial diversity of isolates of *P. infestans* characterized by normalized Shannon diversity index (*h*_0’_) in potato breeding fields in Estonia during 2001–2007 and 2010–2014.

Year	*h*_0’_
2001	0.79
2002	0.91
2003	1.00
2004	0.77
2005	0.69
2006	0.68
2007	0.69
2010	0.95
2011	0.78
2012	0.60
2013	0.43
2014	0.57
Grand Total	0.66

**Table 3:** Year of sampling, potato (*Solanum tuberosum*) cultivars studied, and number of *Phytophthora infestans* isolates tested for mating type, metalaxyl resistance, and virulence for strains collected from the potato breeding fields in Estonia during periods 2001–2007 and 2010–2014.

		Isolate Number Tested for		
Year	Potato Breeding Lines/Cultivars	Mating Type	Metalaxyl Resistance	Virulence
2001	Breeding lines (359, 386, 476, 477, 569, 1370-94, 1572-98, 458-98, 522-98, 93-BXY-1)/Cultivars (Ando, Anti, Ants, Danva, Folva, Impala, Kuras, Latona, Oleva Sarme, Sava, Van Gogh)	25	31	35
2002	Breeding lines (391-93, 405-98, 92-BVU-2, 93-BXL-11, R437-98, R989-93, R992-95)/Cultivars (Ando, Anti, Ants, Asterix, Danva, Kuras, Maret, Ofelia, Oleva, Piret, Sante, Sarme)	13	41	41
2003	Cultivars (Ants, Berber, Bintje, Folva, Latona, Oleva, Sarme, Van Gogh)	9	14	14
2004	Cultivars (Bintje, Fresco, Impala, Latona, Milva, Piret, Platina, Remarka, Agrie dzeltenie/Varajane kollane, Victora)	18	18	19
2005	Cultivars (Alpha, Anti, Ants, Evita, Juku, Oleva, Picasso, Piret, Raja, Sarme)	17	17	18
2006	Cultivars (Ando, Anti, Ants, Asterix, Berber, Granola, Juku, Maret, Princess, Sante, Sarme, Satina, Sinora, Van Gogh)	30	29	30
2007	Cultivars (Ando, Anti, Ants, Fontane, Juku, Latona, Maret, Secura, Agrie dzeltenie/Varajane kollane)	21	21	23
2010	Breeding lines (R1067-05, R3456-06; R458-07)/Cultivar (Asterix)	11	8	11
2011	Breeding lines (R1003-05, R3456-06)/Cultivar (Anti)	21	16	21
2012	Cultivars (Anti, Certo, Evolution, Agrie dzeltenie/Jõgeva Kollane, Sarme)	16	14	15
2013	Cultivars (Ambition, Arielle, Birgit, Evolution, Milva, Rosella)	12	12	12
2014	Breeding line (127-12)/Cultivars (Arielle, Flavia, Red Lady, Solist)	14	14	12
Total		207	235	251

## Data Availability

Not applicable.

## Supplementary Materials

The following supporting information can be downloaded at: https://www.mdpi.com/article/10.3390/plants11182426/s1, Figure S1: Changes in the prevalence of six most common virulence races in *Phytophthora infestans* populations collected from Estonian potato (*Solanum tuberosum*) breeding fields over the study period 2001–2014. Figure S2: Correlations of racial diversity of *P. infestans* isolates and late blight resistance category of potato cultivar in potato breeding fields in Estonia for 2001–2014. Data were fitted by linear regressions; Table S1: Resistance of potato (*Solanum tuberosum*) foliage to late blight. Table S2: Number of different pathotypes among isolates of *Phytophthora infestans* from potato breeding fields in Estonia (2001–2007, 2010–2014).
