# Supplementary material for: High Temporal Variability in Late Blight Pathogen Diversity, Virulence, and Fungicide Resistance in Potato Breeding Fields: Results from a Long-Term Monitoring Study
Source: Plants (Basel). 2022 Sep 16;11(18):2426. doi: 10.3390/plants11182426 (PMC9502785; doi:10.3390/plants11182426)
Supplement: Supplementary file 1 [file plants-11-02426-s001.zip › plants-1883639-supplementary.pdf]

Supplementary Materials

Supplementary Table S1. Resistance to late blight on foliage.

Cultivar/Breeding line	Cultivar resistance	Number of isolates
Alpha	moderately resistant[a]	2
Ambition	susceptible[b]	1
Ando	resistant[c]	9
Anti	resistant	32
Ants	resistant	11
Arielle	susceptible	5
Asterix	susceptible	6
Berber	susceptible	4
Bintje	susceptible	3
Birgit	susceptible	2
Certo	susceptible	3
Danva	susceptible	4
Evita	moderately resistant	1
Evolution	susceptible	1
Flavia	susceptible	2
Folva	susceptible	2
Fontane	susceptible	3
Fresco	susceptible	1
Granola	moderately resistant	2
Impala	moderately resistant	3
Juku	resistant	5
Jõgeva kollane	moderately resistant	3
Kuras	resistant	7
Latona	moderately resistant	9
Maret	moderately resistant	7
Milva	moderately resistant	5
Ofelia	moderately resistant	1
Oleva	moderately resistant	6
Picasso	moderately resistant	1

Piret	resistant	6
Platina	moderately resistant	3
Princess	susceptible	2
Raja	moderately resistant	1
Red Lady	moderately resistant	3
Remarka	resistant	1
Rosella	susceptible	4
Sante	susceptible	3
Sarme	resistant	13
Satina	susceptible	5
Sava	moderately resistant	1
Secura	susceptible	1
Sinora	moderately resistant	5
Solist	susceptible	3
Van Gogh	moderately resistant	4
Agrie dzeltenie/Varajane kollane	susceptible	4
Victoria	moderately resistant	2
Vita	moderately resistant	1
359	moderately resistant	3
386	moderately resistant	1
476	moderately resistant	1
477	moderately resistant	2
569	resistant	3
127-12	moderately resistant	3
1370-94	moderately resistant	2
1572-98	resistant	3
391-93	moderately resistant	1
405-98	moderately resistant	1
458-98	moderately resistant	1
522-98	moderately resistant	4
899-97	resistant	1
92-BVU-2	resistant	2
93-BXL-11	moderately resistant	1

93-BXY-1	resistant	1
R 1003-05	resistant	9
R 1067-05	resistant	1
R 3456-06	resistant	5
R 437-98	resistant	1
R 458-07	resistant	4
R 989-93	resistant	2
R 992-95	resistant	1

note - [a] susceptible (very low, very low to low, low); [b] moderately resistant (low to medium, medium, medium to high); [c] resistant (high, high to very high, very high)

Supplementary Table S2. Number of different pathotypes among isolates of *Phytophthora infestans* from potato breeding fields in Estonia (2001–2007, 2010–2014).

Year of isolation	Pathotype	Number of virulence factors	Number of isolates	Percentage
2001	1.2.3.4.7.8.10.11	8	5	14
	1.2.3.4.6.7.8.9.10.11	10	3	8
	1.2.3.4.6.7.9.10.11	9	3	8
	1.2.3.4.7.10.11	7	3	8
	1.2.3.4.5.6.7.9.10.11	10	2	6
	1.2.3.4.6.7.10.11	8	2	6
	1.2.4.6.7.9.10.11	8	2	6
	1.3.4.6.7.8.10.11	8	2	6
	1.3.4.6.7.8.9.10.11	9	2	6
	1.3.4.7.8.10.11	7	2	6
	Pathotypes found once		9	26
2002	1.2.3.4.6.7.10.11	8	3	7
	1.2.3.4.6.7.8	7	2	5
	1.2.3.6.7.8.10.11	8	2	5
	1.3.4.6.7.10	6	2	5
	1.3.4.6.7.8.10	7	2	5
	1.3.4.6.7.8.10.11	8	2	5
	1.3.4.7	4	2	5
	1.3.4.7.10.11	6	2	5
	1.3.7.10	4	2	5
	Pathotypes found once		22	53

2003	Pathotypes found once		14	100
2004	1.2.3.4.7.10.11	7	5	26
	1.2.3.4.7.8.10.11	8	3	16
	1.2.3.4.6.7.8.9.10.11	10	2	11
	Pathotypes found once		9	47
2005	1.2.3.4.5.6.7.10.11	9	4	22
	1.2.3.4.5.6.7.8.9.10.11	11	3	17
	1.2.3.4.6.7.8.10.11	9	3	17
	1.2.3.4.5.7.8.10.11	9	2	11
	1.2.3.4.6.7.10.11	8	2	11
	1.2.3.4.7.8.10.11	8	2	11
	Pathotypes found once		2	11
2006	1.2.3.4.6.7.8.10.11	9	7	23
	1.2.3.4.6.7.10.11	8	6	20
	1.3.4.7.10.11	6	4	13
	1.3.4.7.8.10.11	7	2	7
	1.4.7.10.11	5	2	7
	Pathotypes found once		9	30
2007	1.2.3.4.7.8.10.11	8	6	26
	1.2.3.4.7.10.11	7	4	17
	1.2.3.4.6.7.10.11	8	3	13
	1.2.3.4.5.6.7.10.11	9	2	9
	1.2.3.4.6.7.8.10.11	9	2	9
	Pathotypes found once		6	26
2010	2.3.7	3	2	18
	Pathotypes found once		9	82
2011	1.2.3.4.6.7.10.11	8	6	29
	1.2.3.4.7.10.11	7	3	14
	Pathotypes found once		12	57

2012	1.2.3.4.6.7.10.11	8	7	47
	1.2.3.4.7.10.11	7	2	13
	2.7.11	3	2	13
	Pathotypes found once		4	27
2013	1.2.3.4.7.8.10.11	8	7	58
	1.2.3.4.6.7.8.10.11	9	3	25
	Pathotypes found once		2	17
2014	1.2.3.4.7.10.11	7	5	42
	1.2.3.4.7.8.10.11	8	3	25
	1.3.4.7.10.11	6	2	17
	Pathotypes found once		2	17

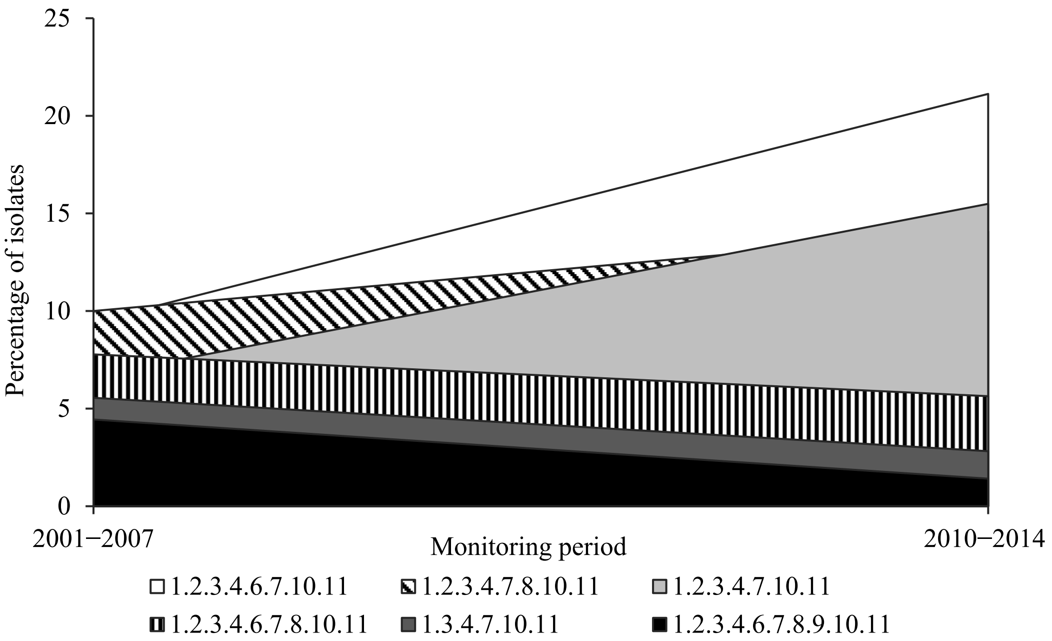

Supplementary Figure S1. Changes in the prevalence of six most common virulence races in *Phytophthora infestans* populations collected from Estonian potato breeding fields over the study period 2001–2014.

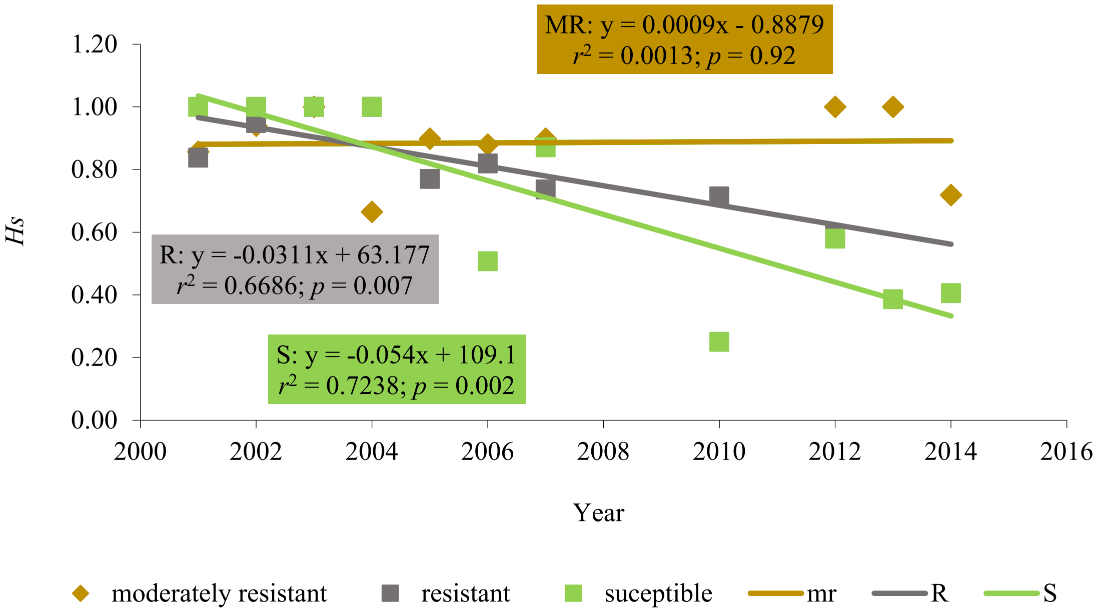

Supplementary Figure S2. Correlations of racial diversity of *P. infestans* isolates and late blight resistance category of potato cultivar in potato breeding fields in Estonia for 2001–2014. Data were fitted by linear regressions.
